# Supplementary material for: Young domestic chicks spontaneously represent the absence of objects
Source: eLife. 2022 Apr 11;11:e67208. doi: 10.7554/eLife.67208 (PMC9000949; doi:10.7554/eLife.67208)
Supplement: Supplementary file 1. [file elife-67208-supp1.docx]

**Supplementary Information**

1. **Looking time data**

| **Experiment** | **Condition** | **Total Mean ± SE** | **Male Mean ± SE** | **Female Mean ± SE** |
| --- | --- | --- | --- | --- |
| Experiment 1 | Expected Disappearance | 12.67 ± 1.26 | 12.18 ± 1.48 | 13.2 ± 2.14 |
|  | Unexpected Disappearance | 15.42 ± 1.41 | 14.27 ± 2.12 | 16.66 ± 1.87 |
| Experiment 2 | Expected Appearance | 14.56 ± 1.77 | 12.24 ± 2.85 | 15.57 ± 2.14 |
|  | Unexpected Appearance | 12.91 ± 1.6 | 10.45 ± 2.14 | 15.03± 2.24 |
| Experiment 3 | Expected Appearance | 16.61 ± 1.59 | 16.08 ± 2.26 | 17.18 ± 2.3 |
|  | Unexpected Appearance | 17.65 ± 1.55 | 18.42 ± 2.14 | 16.82 ± 2.3 |
| Experiment 4 | Expected Appearance | 13.03 ± 1.86 | 12.29 ± 2.6 | 15.27 ± 2.70 |
|  | Unexpected Appearance | 13.55 ± 1.66 | 14.54 ± 2.56 | 12.60 ± 2.15 |

**Supplementary table 1**. **Average values of looking time data in Experiment 1-4**. We report the mean values and standard errors of looking times for the total sample size, and separately for males and females in the four experiments.

We run a repeated measures ANOVA on the square root transformed looking time data of Experiment 3 and 4. A 2x2 repeated measures ANOVA with Outcome (Expected vs. Unexpected) and Sex (Female vs. Male) as factors did not yield any significant effects.
